# Analysis of trends and hotspots in the research on sleep disorders of maintenance hemodialysis patients based on bibliometrics

**DOI:** 10.3389/fpsyt.2025.1515516

**Published:** 2025-06-30

**Authors:** Li Huang, Siyu Li, Jing Zeng

**Affiliations:** School of Nursing, Chengdu Medical College, Chengdu, China

**Keywords:** hemodialysis, sleep disorders, bibliometrics, visualization analysis, research hotspots

## Abstract

**Objective:**

To explore the research hotspots and frontiers of sleep disorders in patients undergoing maintenance hemodialysis (MHD) over the past 15 years.

**Methods:**

Relevant literature published from January 1, 2010, to April 1, 2025, was retrieved from the Web of Science Core Collection database. Bibliometric analysis and VOSviewer software were employed to visualize data on countries, institutions, journals, authors, and keywords.

**Results:**

A total of 337 articles were included. The annual publication volume demonstrated an upward trend. China ranked first in contributions (21.3%), followed by the United States (16.9%) and Turkey (12.8%). BMC Nephrology was the most active journal, and the University of Pittsburgh emerged as the leading institution. One major author collaboration group was identified. Research hotspots focused on specific sleep disorders (e.g., sleep apnea, restless legs syndrome), clinical outcomes (e.g., mortality, prevalence), influencing factors, quality of life, and interventions. Emerging trends included psychological and physical comorbidities such as depression, anxiety, pain, and fatigue.

**Conclusion:**

Bibliometric analysis combined with visualization tools effectively maps the current landscape and frontiers of sleep disorder research in MHD patients. These findings provide evidence-based references for future nursing practices and interdisciplinary studies in this field.

## Introduction

End-stage renal disease (ESRD), a rapidly growing public health concern, imposes significant economic burdens on patients’ families and society (1). Maintenance hemodialysis (MHD), the primary renal replacement therapy and final outcome for progressive kidney diseases (2), is associated with an 85% incidence of sleep disorders (3, 4). Sleep disorders are defined as disruptions in sleep quality, duration, or timing, including abnormal physiological events during sleep-wake transitions (5). In MHD patients, these disorders exacerbate quality of life, elevate cardiovascular risks, impair cognition, and increase all-cause mortality (6, 7). Contributing factors span sociodemographic (e.g., age, income), clinical (e.g., dialysis modality, comorbidities), psychological (e.g., anxiety), and environmental domains (8). Effective management may reduce morbidity and mortality while enhancing well-being.

In recent years, sleep disorders in maintenance hemodialysis (MHD) patients have received increasing attention from researchers. Relevant review articles mainly discuss the influencing factors, adverse consequences, and intervention measures. However, few studies have used visualization tools to intuitively reflect the research hotspots and trends of sleep disorders in MHD patients. Mastering the research status quo, hotspots, and overall research direction of sleep disorders in MHD patients is helpful for sorting out the relevant research context and providing a reference for the further development of clinical research and evidence-based practice. Bibliometric analysis is an important method for comprehensively understanding the research progress in a specific scientific data field (9). Compared with traditional review articles, bibliometric analysis explores the expertise in related fields by identifying the research trends of specific topics (10). VOSviewer, developed by Leiden University in the Netherlands, is a software tool focused on the visualization of scientific knowledge. It has mature technology and high reliability and is widely used in bibliometric research (9). VOSviewer has an intuitive graphical display mode and a simple and clear layout. When analyzing the literature in the specific field of sleep disorders in MHD patients, it can clearly identify the main research directions and hot issues, avoiding the impact on the analysis due to overly complex maps. VOSviewer has outstanding advantages in co-word analysis and effective cluster analysis. It can group related literatures, authors, or keywords into different categories. It can classify research from different perspectives and provide a clear framework for in-depth discussion of research hotspots and trends. This study is based on the Web of Science (WOS) Core Collection database and conducts a bibliometric analysis of the literature related to sleep disorders in MHD patients. It intuitively presents the research status quo and hotspots in this field, reveals the development trends of future research, and helps researchers accurately grasp the latest research direction in this field.

## Data and methods

### Data sources and search strategy

**Figure 1:**
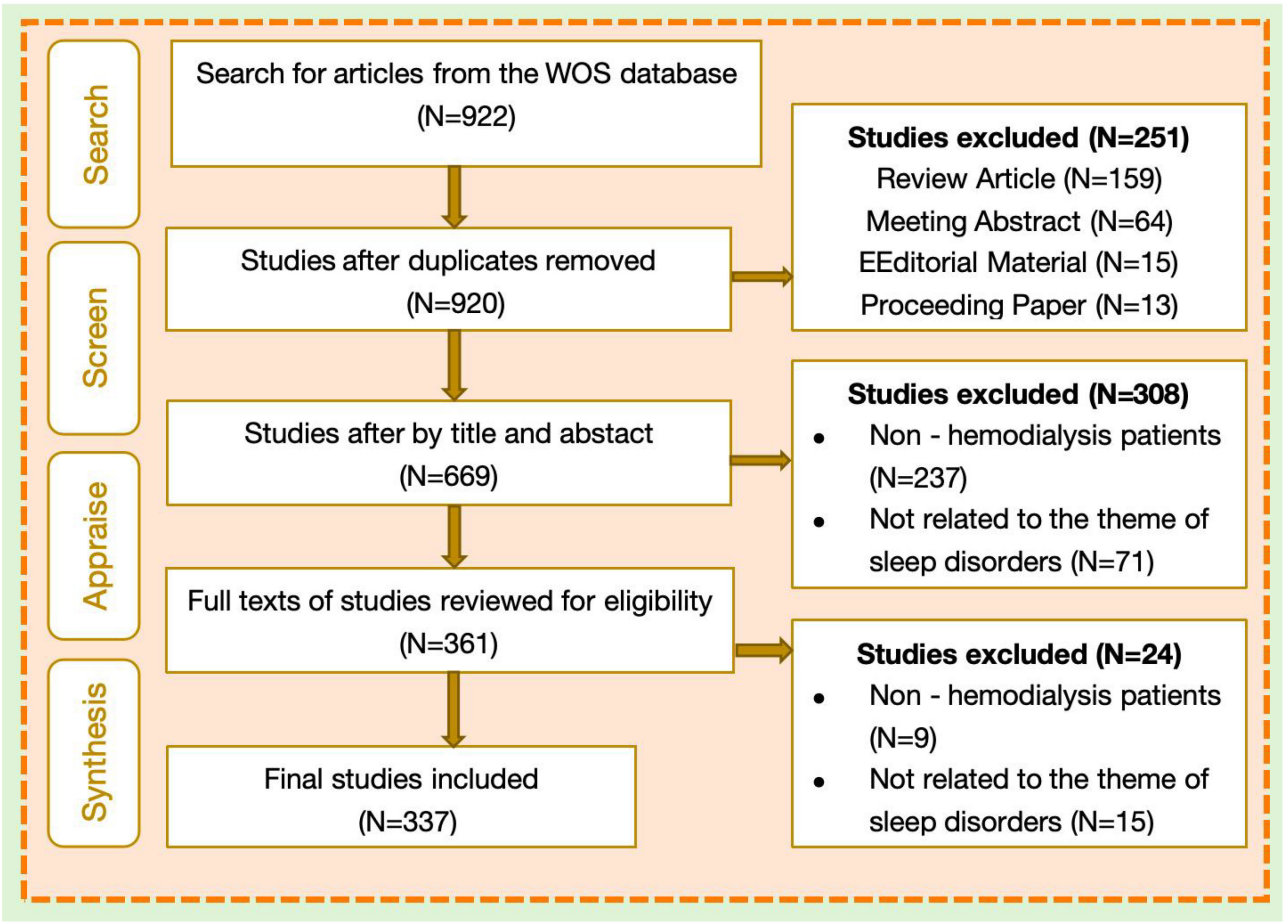
Literature retrieval flow chart.

This study used the Web of Science Core Collection database as the source of articles. The choice is mainly because of the following considerations (11, 12). Firstly, the journals included in this database have been screened through a strict quality evaluation system, which can ensure the authority and academic value of the literature. Secondly, its interdisciplinary coverage highly coincides with the cross - disciplinary characteristics of this study, which involves multiple fields such as medicine, nursing, and neuroscience. Thirdly, the powerful bibliometric analysis functions of the Web of Science (such as co - citation analysis and keyword clustering) can effectively mine research hotspots and trends. Although other databases (such as PubMed and Scopus) also contain relevant literature, considering the accuracy of the research objectives and the adaptability of the analysis tools comprehensively, this study selected the Web of Science as the core data source. The search formula was: (TS=((“sleep disorder*” OR somnipathy) OR insomnia OR “sleep dysfunction” OR “sleep disturbance” OR “sleep quality” OR “sleep deprivation” OR (“night terror*” OR parasomnia) OR hypersomnia OR “restless legs syndrome” OR “sleep apnea”)) AND TS=(“maintenance hemodialysis” OR hemodialysis OR MHD OR hemodialysis)). Publications of article type from January 1, 2010, to April 1, 2025, were included. EndNote software facilitated initial deduplication, followed by manual verification. Two independent reviewers screened the literatures and cross-checked the screening results to ensure the accuracy of the screening. In case of any discrepancies, they would be resolved through discussion or consultation with a third expert. A final set of 337 articles was included; the selection process is detailed in **Figure 1**.

### Research methods

Based on the WOS database, this study performed a bibliometric analysis on the research hot spots and trends of sleep disorders in maintenance hemodialysis patients. According to the keyword field information extracted by the VOS viewer software, we conducted statistics on the keyword frequency, sorted them in descending order, and extracted the high-frequency keywords. We carried out co-occurrence clustering visual analysis on these high-frequency keywords. In the keyword co-occurrence clustering map, each round node represents a keyword, while the size of the node depends on the keyword’s frequency. A connection line represents the co-occurrence relationship between the two connected keywords, and the thickness and length of the connection line are positively and negatively correlated with the correlation strength between them, respectively. The color (region) of the node represents the cluster to which it belongs. At the same time, the paper analyzed the co - occurrence timeline of high-frequency keywords. In the keyword co - occurrence timeline map, different colors represent different years in which keywords appeared. The blue node color (in the lower position) represents an earlier appearance time of keywords, and the yellow node color (in the upper position) represents a higher degree of keyword emergence.

## Results

### Literature output

#### Annual distribution of publications

**Figure 2:**
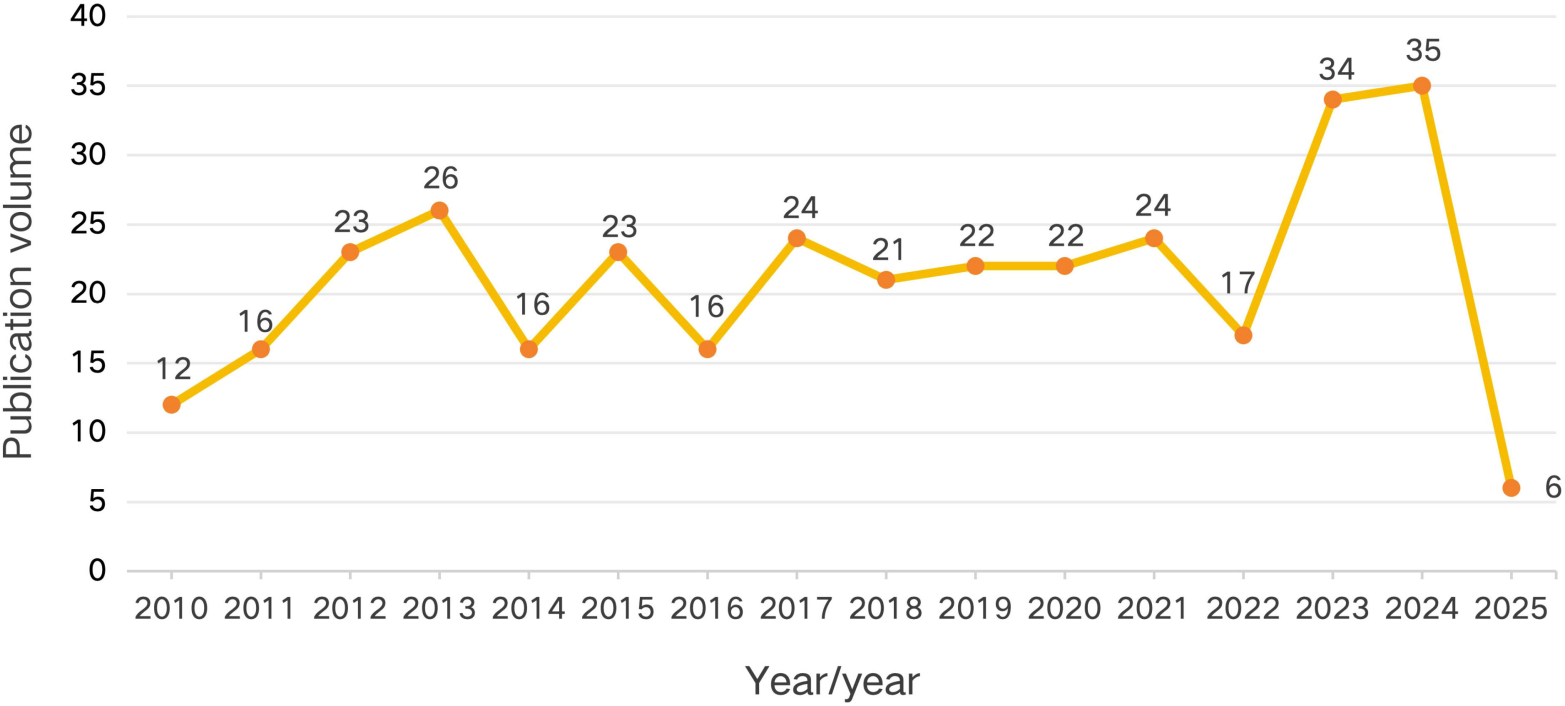
Time-series of publication volume in the research field of MHD sleep disorder.

Over the past 15 years, the annual number of published papers in the research field of sleep disorders in MHD patients has generally shown an upward trend. In 2010, the initial number of published papers was 12, and then the quantity gradually increased, reaching 26 in 2013. There were fluctuations from 2014 to 2018, and the number of published papers remained relatively stable from 2019 to 2021. In 2023 and 2024, there was a significant increase, with the number of published papers being 34 and 35 respectively. In 2025, the number decreased to 6, which may be related to the fact that the literature retrieval was conducted up to April 1st. Despite these fluctuations, the upward trend highlights the growing research attention paid to this field. As shown in **Figure 2**.

#### Journal distribution

In the field of sleep disorders in MHD patients, a bibliometric analysis was conducted to explore the distribution of journals. As shown in **Table 1**, Bmc Nephrology is the journal that has published the largest number of articles. A total of 16 articles have been published in this journal. Hemodialysis International ranks second, with 12 articles published, followed by International Urology and Nephrology, which has published 11 articles. These journals mainly focus on research related to the field of nephrology and sleep medicine.

**Table 1:** Top 10 journals by publication volume (2010-2025).

Ranking	Journal	Articles
1	*Bmc Nephrology*	16
2	*Hemodialysis International*	12
3	*International Urology and Nephrology*	11
4	*Sleep Medicine*	10
5	*Clinical Journal of the American Society of Nephrology*	10
6	*Nephrology Dialysis Transplantation*	9
7	*Renal Failure*	8
8	*Therapeutic Apheresis and Dialysis*	7
9	*Sleep and Breathing*	7
10	*Blood Purification*	7

#### Country distribution

The country distribution analysis is based on the publication data from the Web of Science Core Collection between 2010 and 2025. The top three countries in terms of the number of published papers are China (21.3%), the United States (16.9%), and Turkey (12.8%). As shown in **Table 2**.

**Table 2:** Top 5 countries in publication volume in WOS (2010-2025).

Ranking	Country	Number of articles issued (articles)	Cited frequency
1	China	72	870
2	United States	57	1568
3	Turkey	43	705
4	Iran	25	449
5	Canada	23	730

#### Institutional distribution

**Figure 3:**
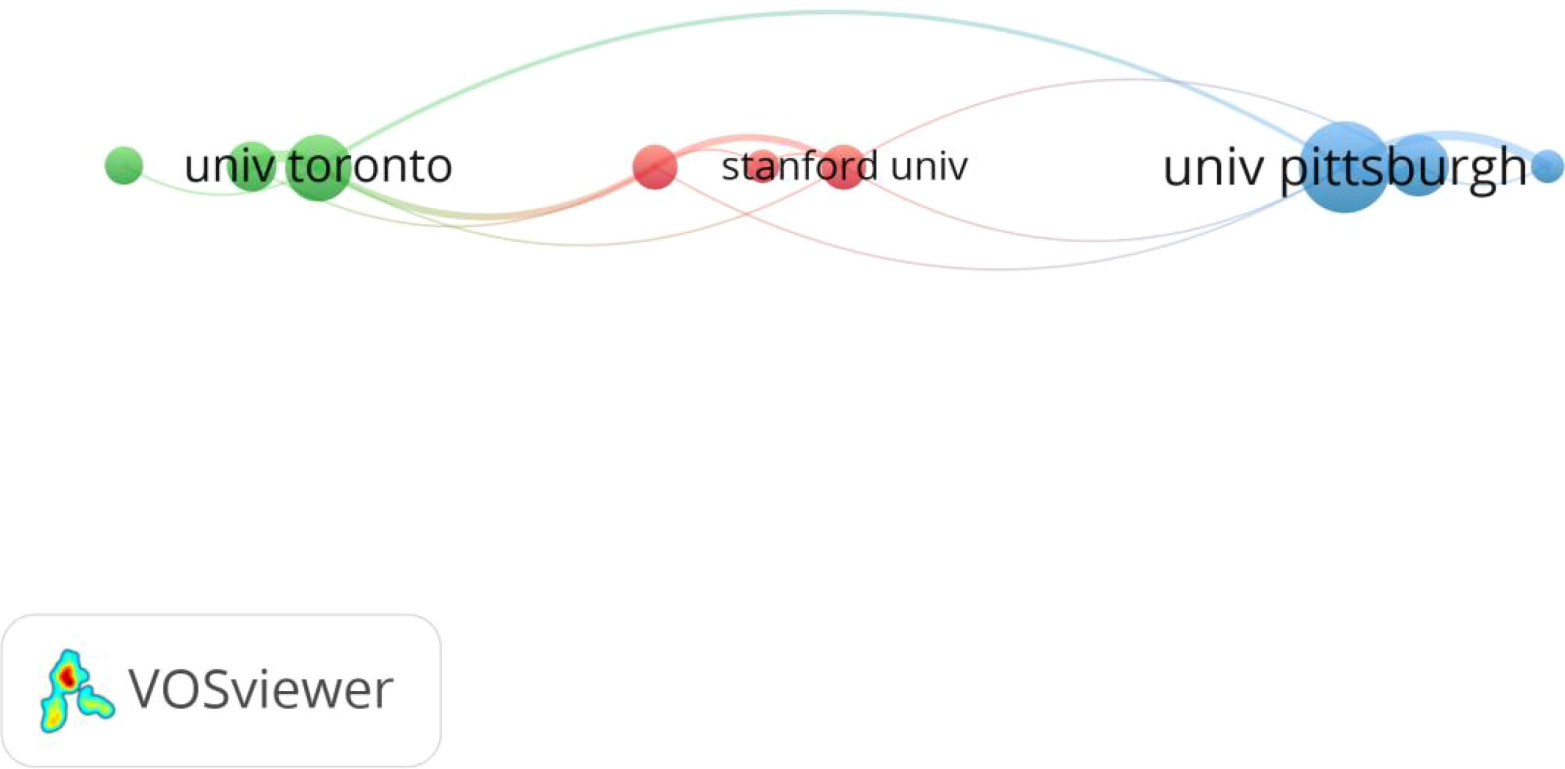
Co-authorship analysis of publishing institutions in MHD patients’ sleep disorder field.

Among the top ten institutions in terms of the number of published papers, the University of Pittsburgh has the highest number of publications (see **Table 3**). As shown in **Figure 3**, through conducting a Co-authorship analysis of the institutions that published papers, it was found that there is a certain correlation among the University of Toronto, Stanford University, and the University of Pittsburgh in the research field of sleep disorders in MHD patients.

**Table 3:** Top 10 institutions in publication volume (2010-2025).

Ranking	Institution	Articles
1	University of Pittsburgh	15
2	University of Thessaly	12
3	University of Toronto	10
4	University of Nicosia	9
5	University of New Mexico	9
6	Selcuk University	8
7	University Health Network	7
8	Capital Medical University	7
9	Nanjing Medical University	7
10	University of California, San Francisco	6

#### Author analysis

**Figure 4:**
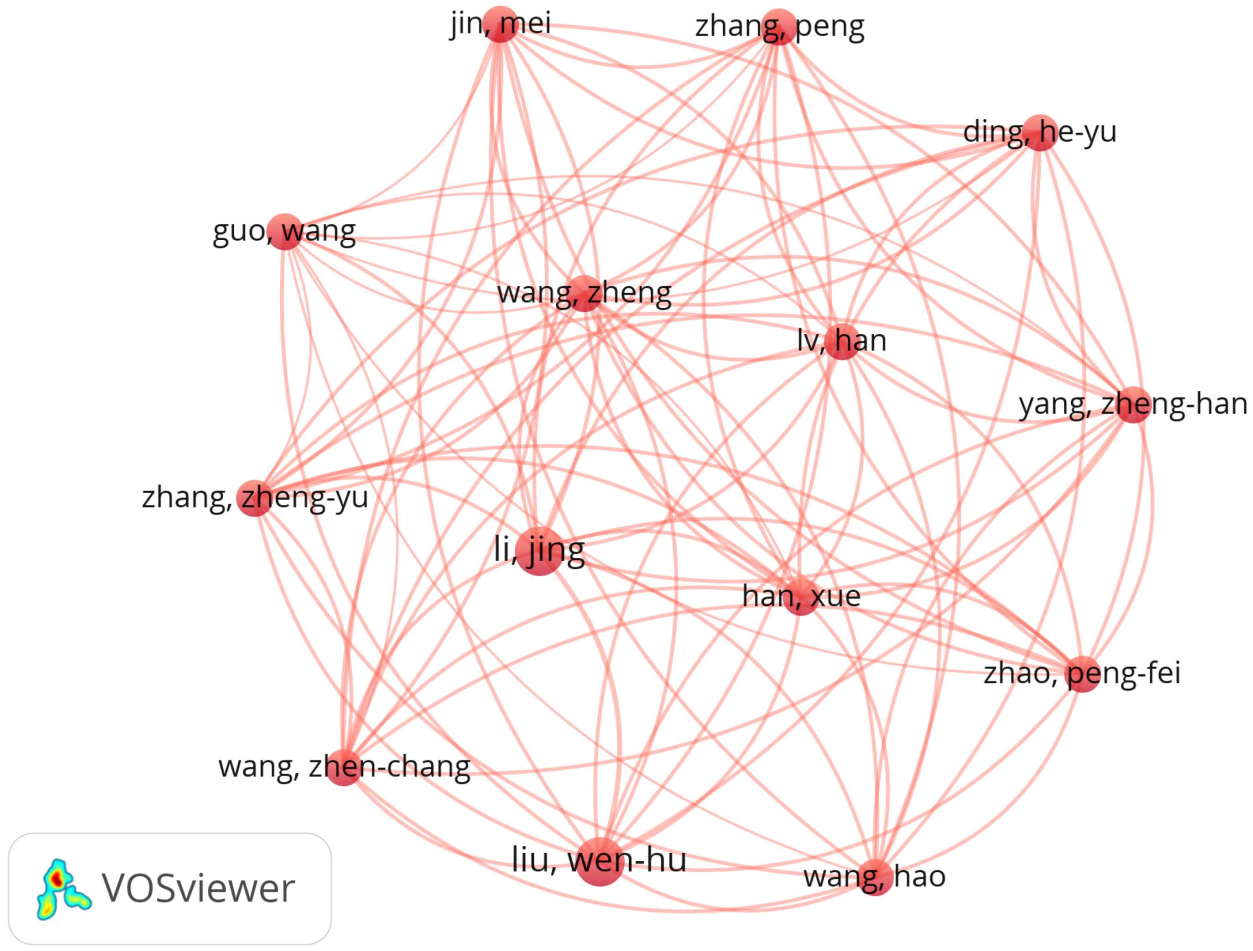
MHD sleep disorder research author collaboration network.

A total of 1,835 authors published on MHD - related sleep disorders. Christoforos D. Giannaki led with 9 papers (**Table 4**). Using Price’s Law (13) (N=0.749×ηmax^1/2^), authors with 3+ publications were classified as core. **Figure 4** shows a co-authorship map of 71 core authors, revealing a major cluster with 14 closely-related key authors.

**Table 4:** Top 5 authors by publication volume (2010-2025).

Ranking	Author	Articles	Cited
1	Christoforos D. Giannaki	9	346
2	Christina Karatzaferi	9	346
3	Giorgos K. Sakkas	9	346
4	Ioannis Stefanidis	9	346
5	Georgios M. Hadjigeorgiou	8	340

### Research trends analysis

#### High-frequency keywords

High-frequency keywords reflect the research hotspots in this field (9). By conducting a keyword co-occurrence analysis using VOS viewer, it was found that there were a total of 1,102 keywords in the literatures retrieved from the WOS. 64 keywords (accounting for 5.8%) with a frequency of occurrence exceeding 10 times were screened out. After excluding disease-related nouns, the top 10 high-frequency keywords are shown in **Table 5**, which mainly include “Quality of life”, “Depression”, “Restless legs syndrome”, “Mortality”, “Sleep apnea”, and so on.

**Table 5:** Top 10 high-frequency keywords in literature (2010-2025).

Ranking	Keyword	Frequency
1	Quality of life	158
2	Depression	95
3	Restless legs syndrome	79
4	Mortality	79
5	Sleep apnea	73
6	Influencing factors	63
7	Insomnia	60
8	Treatment	33
9	Daytime sleepiness	31
10	Anxiety	29

#### Clustering analysis

**Figure 5:**
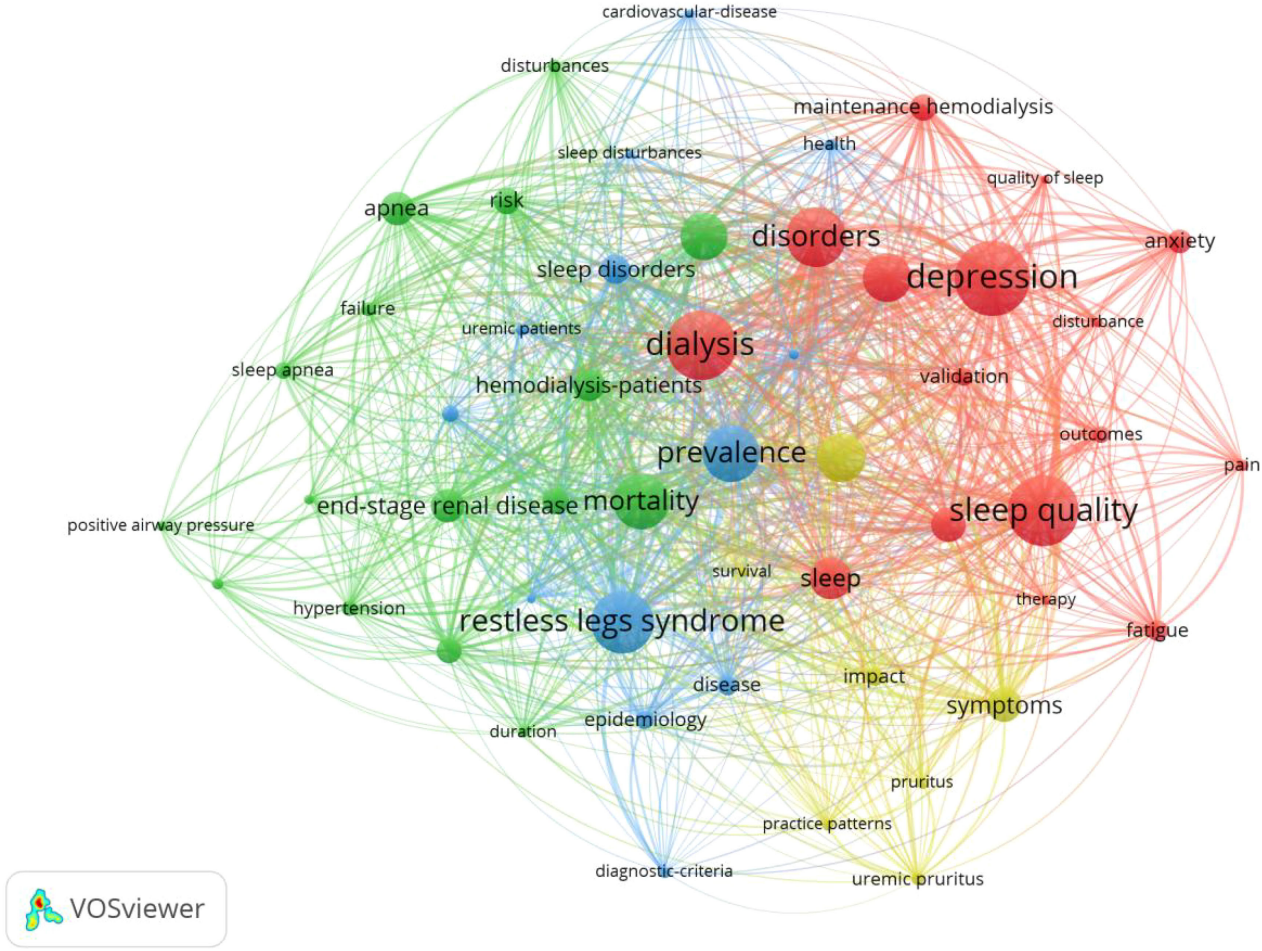
Keyword co-occurrence cluster map of international MHD sleep disorder research (2010-2025).

Keywords that appeared 10 times or more were selected to draw a clustering map of keyword co-occurrence. The literatures retrieved from the WOS database have highlighted four color clustering clusters: 1)The red clustering cluster contains keywords such as “depression”, “anxiety”, “pain”, and “fatigue”, highlighting the close association between psychological and physical discomfort factors and sleep disorders in this patient group. 2)The green clustering cluster includes keywords like “prevalence”, “mortality”, “sleep apnea”, and “risk”, focusing on aspects related to epidemiology and health outcomes. 3)The blue clustering cluster contains “restless legs syndrome”, “duration”, “disease”, and “impact”, delving into specific sleep-related conditions and their impacts. 4)The yellow clustering cluster has keywords related to sleep-related symptoms and “itching”, centering around the manifestations of sleep problems and related symptoms. As shown in **Figure 5**.

#### Temporal overlay analysis

**Figure 6:**
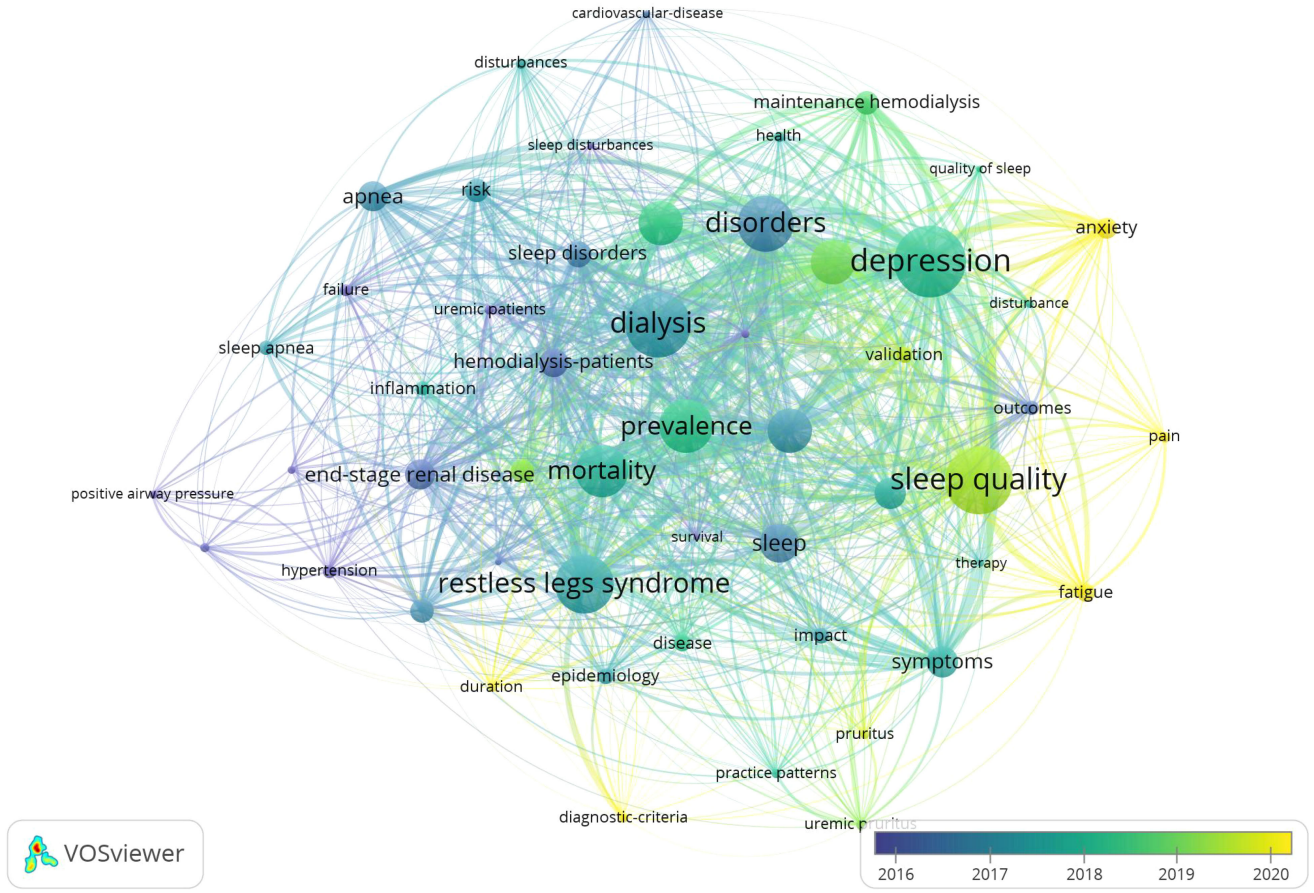
Temporal overlay visualization of international MHD sleep disorder research keywords (2010-2025).

Through the visualization analysis with year overlay of the WOS literatures in the research field of sleep disorders in MHD patients, it was found that: Specific manifestations of sleep disorders such as sleep apnea and restless legs syndrome, outcome indicators like mortality and prevalence, influencing factors, quality of life, and treatment methods have always been the key focuses in this field. As time goes by, psychological and physical discomfort factors closely associated with sleep disorders, such as depression, anxiety, pain, and fatigue, have become the research hotspots in this field in recent years. As shown in **Figure 6**.

## Discussion

In this study, by using the method of literature metrology, we analyzed the number of articles published annually, the distribution of journals, the distribution of publishing institutions, the high-frequency keyword analysis, the keyword co-occurrence clustering and the visual analysis of the timeline, and comprehensively analyzed and summarized the research results related to MHD sleep disorders, which would help researchers to understand the current situation, trends and hot spots of research in this field.

### Current research status of MHD sleep disorders

Currently, countries around the world maintain a steady level of attention to the research on sleep disorders in MHD patients, and there is still considerable room for in-depth research in this field in the future. In terms of the number of published articles, from 2010 to 2025, the overall number of published articles in this field showed an upward trend. In particular, the number of published articles increased relatively rapidly in 2023 and 2024. China has the largest number of published articles, which may be due to the continuous increase in the total number of patients receiving hemodialysis in China. According to the report of the Chinese Renal Data System (CNRDS) (14), as of the end of 2023, the number of hemodialysis patients in China reached 916,000. Regarding the publishing institutions, the University of Pittsburgh is in a leading position, indicating that the researchers of this institution have continuous and in-depth attention to the sleep disorder problems of hemodialysis patients. From the perspective of the publishing teams and institutions, the University of Pittsburgh, the University of Toronto, and Stanford University have established corresponding cooperative connections, suggesting that future research can draw on the multidisciplinary research model of this team and integrate the advantageous resources in the fields of sleep medicine, nephrology, and other related fields. It is recommended that scientific research teams from various countries actively participate in international cooperation, establish a global academic exchange platform, jointly develop personalized intervention programs for patients of different ethnic groups and regions, promote the clinical transformation and application of research achievements in this field, and ultimately improve the sleep quality and survival prognosis of MHD patients.

### Research hot spots and frontier analysis of MHD sleep disorder

Specific manifestations related to sleep disorders in MHD patients, such as sleep apnea and restless legs syndrome, outcome indicators like mortality and prevalence, influencing factors, and quality of life have always been the key focuses in this field. As for the psychological and physical discomfort symptoms associated with sleep disorders, such as depression, anxiety, pain, and fatigue, they represent the new research trends in this field. The following is a summary of the research hotspots and new trends in this field.

### Psychosomatic discomfort symptoms related to sleep disorders

In recent years, research on the psychological and physical discomfort symptoms associated with sleep disorders has gradually become a new trend in the field of sleep disorders in MHD patients. There is a complex two-way interaction relationship between sleep disorders and psychosomatic discomfort symptoms. In terms of psychology, due to long-term renal failure in MHD patients, the accumulation of toxins in the body and metabolic disorders are likely to trigger sleep structure disorders. This, in turn, can lead to an imbalance of neurotransmitters in the brain and induce emotional disorders such as depression and anxiety (15). Moreover, the states of anxiety and depression can further disrupt the sleep rhythm, forming a vicious cycle. Many research analyses have pointed out that anxiety and depression are independent predictors of poor overall sleep quality (16, 17). In terms of the body, as common somatic symptoms, pain and fatigue also interact with sleep disorders. Chronic pain can interfere with patients’ ability to fall asleep and maintain sleep. On the other hand, long-term lack of sleep can lower the pain threshold, making the perception of pain more intense and increasing the sense of fatigue (18, 19). In the future, research in this field can further explore personalized intervention strategies. Since there are differences in the types of sleep disorders and the combinations of psychosomatic discomfort symptoms among different patients, a precise assessment system can be established. Personalized treatment plans can be formulated according to the specific conditions of patients. In addition, efforts should also be made to enhance patients’ self-management ability to promote the long-term control of the disease, which will provide a more effective path for improving the overall health status of MHD patients.

### The main manifestations of sleep disorders

Sleep disorders in MHD patients not only have a high incidence rate but also present in various forms, including insomnia, sleep apnea, restless legs syndrome (RLS), etc., and the severity varies from person to person. In this study, insomnia is a high-frequency keyword in this field, and sleep apnea and restless legs syndrome are research hotspots in this field. RLS mainly causes discomfort in the deep muscles of the legs, which worsens during nighttime rest or the quiescent period. Patients need to relieve the symptoms by continuously patting their legs and moving their lower limbs (20). RLS not only leads to impaired sleep quality in MHD patients but is also associated with a significantly higher risk of the incidence and mortality of cardiovascular diseases (21), which urgently requires the attention of clinical medical staff. In the future, intervention measures should be reasonably selected according to the individual disease conditions of patients to relieve the discomfort symptoms of their limbs and improve their sleep quality. The prevalence of sleep apnea in hemodialysis patients is as high as 57% (22). It is closely related to the occurrence and development of diseases such as cardiovascular diseases, diabetes, right heart failure, and polycythemia (23). Some studies have shown that sleep apnea may exacerbate the micro-inflammatory state of hemodialysis patients and accelerate the disease progression (24). This reminds us that for MHD patients with sleep apnea, appropriate parameters for positive airway pressure ventilation treatment or surgical intervention measures should be selected in a timely manner according to the severity of their conditions, and long-term follow-up and management of patients should be strengthened.

### Adverse outcomes

Long-term sleep disorders will seriously affect the physical and mental health of patients with MHD, and are likely to trigger adverse emotions, reducing the patients’ daily living ability. This will further exacerbate the progression of the disease, affect the prognosis of the disease, severely impact the patients’ quality of life, and increase their mortality rate. Some studies have shown that sleep disorders not only seriously affect the quality of life of MHD patients, but also lead to sleep deprivation, which disrupts the patients’ normal circadian rhythm. As a result, there is a significant decline in the body’s immunity, making them highly susceptible to hypertension and hyperglycemia. This significantly increases the incidence of cardiovascular risk events and all-cause mortality in patients, indicating that the severity of sleep disorders may directly influence the prognosis and quality of life of MHD patients (25, 26). After a 12-month investigation, Wang Jialin (27) and his colleagues analyzed MHD patients and found that 52% of the patients had poor sleep quality. Moreover, those patients with poor sleep quality also had a lower quality of life and a higher mortality rate. This suggests that future researchers should pay more attention to the serious adverse consequences brought about by sleep disorders.

### Influencing factors

The incidence of sleep disorders in MHD patients is relatively high, and the influencing factors are a hot topic in the research of sleep disorders. There are many factors that can affect sleep disorders in maintenance hemodialysis (MHD) patients, and factors in physiological, social, and psychological aspects may all play a role. The disease itself and its complications can affect sleep quality. Some studies have shown that comorbidities can serve as an independent factor for predicting sleep disorders. The more comorbidities a patient has, the more likely it is that their sleep quality will be unsatisfactory (28). Previous studies have found that sleep disorders in MHD patients are closely related to serum indices such as hemoglobin, calcium, phosphorus, and thyroid hormones (29, 30). Social demographic factors also have an impact on the sleep quality of MHD patients. Among them, age is considered to be one of the important factors affecting sleep disorders (31). In addition, economic level, as well as family and social support, are also closely related to sleep quality (32). Sleep disorders are a complex problem influenced by the interaction of multiple factors. The dominant influencing factors of sleep disorders vary among different MHD patients. Future research can pay full attention to individual differences and formulate personalized intervention plans for each patient. By comprehensively considering the patient’s physiological, psychological, and social dimensions, individual factors can be further analyzed in depth to better meet the patients’ needs.

### Quality of life

This study shows that the quality of life of patients with MHD sleep disorders is a research hotspot in this field. Long-term sleep disorder will seriously affect the physical and mental health of patients with MHD, and are prone to produce adverse emotions such as anxiety and depression, affecting the patients’ ability to live daily, reducing confidence in the treatment of the disease, further aggravating the development of the disease, and seriously affecting the quality of life of patients (33, 34). Wang Jialin et al. (27) analyzed 112 cases of MHD patients after 12 months of investigation, and found that 52% of the patients had poor sleep quality, and the quality of life of those with poor sleep quality was generally poor. Li Jiuhong et al. (35) evaluated and analyzed 143 patients with MHD, and found that these patients had many serious symptoms, 88% of them had sleep disorder, and the scores of various dimensions of quality of life were generally low, suggesting that the quality of life of patients was poor. Multiple studies have shown that for MHD patients with sleep disorders, the scores in all dimensions of quality of life are generally low, the quality of life of patients is poor, and the mortality rate is higher (31, 36). Timely recognition and treatment of sleep disorders in MHD patients can improve the quality of life and disease prognosis.

## Conclusion

This study employed a visual statistical method to systematically sort out the relevant literatures in the field of sleep disorders in MHD patients over the past 15 years, and presented the research status quo, hot spot distribution, and frontier development trends in this field in an intuitive manner. During the research process, we extensively collected literature materials through various channels and used scientific and rigorous analysis methods to draw relatively objective and reliable conclusions. However, limited by the research conditions and methods, this study still has certain limitations. Firstly, this study only selected the Web of Science database as the data source, and there were certain restrictions in terms of the language of the literatures. It failed to fully cover the relevant research achievements in other languages. Therefore, it is recommended that future research can further expand the data sources, incorporate more authoritative databases, and at the same time break through the language barrier, comprehensively collect literatures in different languages, so as to explore the overall picture of the research in this field more comprehensively and deeply. Future research can combine methods such as expert review and field research to explore potential novel research directions.

## Data Availability

The raw data supporting the conclusions of this article will be made available by the authors, without undue reservation.
